# ASTool: An Easy-to-Use Tool to Accurately Identify Alternative Splicing Events from Plant RNA-Seq Data

**DOI:** 10.3390/ijms23084079

**Published:** 2022-04-07

**Authors:** Huan Qi, Xiaokun Guo, Tianpeng Wang, Ziding Zhang

**Affiliations:** State Key Laboratory of Agrobiotechnology, College of Biological Sciences, China Agricultural University, Beijing 100193, China; qihuanv587@163.com (H.Q.); xiaokunguo@cau.edu.cn (X.G.); tpengwang@163.com (T.W.)

**Keywords:** plant, alternative splicing, RNA-Seq data, bioinformatics

## Abstract

Alternative splicing (AS) is an essential co-transcriptional regulatory mechanism in eukaryotes. The accumulation of plant RNA-Seq data provides an unprecedented opportunity to investigate the global landscape of plant AS events. However, most existing AS identification tools were originally designed for animals, and their performance in plants was not rigorously benchmarked. In this work, we developed a simple and easy-to-use bioinformatics tool named ASTool for detecting AS events from plant RNA-Seq data. As an exon-based method, ASTool can detect 4 major AS types, including intron retention (IR), exon skipping (ES), alternative 5′ splice sites (A5SS), and alternative 3′ splice sites (A3SS). Compared with existing tools, ASTool revealed a favorable performance when tested in simulated RNA-Seq data, with both recall and precision values exceeding 95% in most cases. Moreover, ASTool also showed a competitive computational speed and consistent detection results with existing tools when tested in simulated or real plant RNA-Seq data. Considering that IR is the most predominant AS type in plants, ASTool allowed the detection and visualization of novel IR events based on known splice sites. To fully present the functionality of ASTool, we also provided an application example of ASTool in processing real RNA-Seq data of *Arabidopsis* in response to heat stress.

## 1. Introduction

In eukaryotes, many precursor mRNAs (pre-mRNAs) contain at least one intron. Alternative splicing (AS) refers to the process of producing different mRNA splicing isoforms from a precursor mRNA by selecting different combinations of splicing sites. AS events in animal genes have been intensively investigated and widely reported. It has been well established that AS was closely related to cell development, cell differentiation, and cell-specific function [1,2]. In particular, abnormality of AS could lead to diverse human diseases [3]. In plants, there is increasing evidence that AS plays an important role in biological timing, development, tissue-specific patterns, and stress response [4,5,6,7]. With the development of RNA sequencing technology, the identification and analysis of AS events from RNA-Seq data have become routine tasks of transcriptome analysis. In the past several years, comprehensive AS analysis of RNA-Seq data has been widely used to understand plant stress responses [8,9,10]. For instance, Vitoriano and Calixto investigated heat stress-induced AS events in rice leaves from publicly available RNA-Seq data [11]. Cecchini et al. found that heat stress-regulated AS affects the subnuclear localization of DNA glycosidase MBD4L in *Arabidopsis* [12]. Martin et al. (2021) constructed a data resource called PastDB to manage AS and gene expression quantification data in *Arabidopsis* and the following large-scale analysis revealed that *Arabidopsis* disproportionately employs AS for the stress responses [13]. In the foreseeable future, accurate identification and comprehensive analysis of AS events based on increasingly available plant RNA-Seq data will still be in high demand, which will accelerate our understanding of the molecular mechanisms of plant AS.

AS events include four main types [14]: intron retention (IR), exon skipping (ES), alternative 5′ splice sites (A5SS), and alternative 3′ splice sites (A3SS). Generally, ES is the most common form in animals [15], but IR is the most prevalent form in plants [16,17,18]. This phenomenon could be explained by the difference in the AS mechanism between plants and animals. The introns of plants tend to be shorter compared to animals, suggesting that the recognition of introns and exons might be different [19]. The model in which the intron is recognized as a unit is called “intron-definition” while the model in which the exon is recognized as a unit is called “exon-definition”. Previous studies suggested that “intron-definition” splicing generally resulted in IR, whereas “exon-definition” splicing led to ES [20].

Existing methods for AS detection can be classified into two groups: exon-based methods and isoform-based methods. Exon-based methods can directly detect the usage of exons, such as MISO [21], rMATS [22], IRFinder [23], and Whippet [24]. For example, IRFinder provides a complete pipeline for identifying IR events, including alignment via the STAR algorithm, IR quantification, and comparison among multiple samples. Whippet allows a rapid and accurate AS identification from RNA-Seq data, which is particularly effective in analyzing complex AS events. Isoform-based methods consider the relative isoform abundance, such as Cufflinks [25], StringTie [26], and Suppa2 [27]. For instance, Suppa2 can estimate the percent spliced in (PSI) of AS events based on the abundance of transcripts, and analyze differential splicing across multiple biological conditions. However, the estimation of isoform abundance contains uncertainty in read assignments when genes contain multiple isoforms. Thus, exon-based methods generally yield more accurate results and seem more popular in the community. To the best of our knowledge, most existing AS detection methods were initially designed to process RNA-Seq data of animals, and their applicability and performance in plants have not been clearly benchmarked.

By fully taking the characteristics of plant introns and flanking exons into account, in this study, we proposed a tool called ASTool for the detection of plant AS events. As an exon-based method, ASTool can directly calculate the PSI values of AS events. By comparison with several existing AS identification tools using simulated plant RNA-Seq data, ASTool showed a fully competitive performance regarding its identification accuracy, consistency, and computational efficiency. To fully present the functionality of ASTool, we also provide a case study of AS identification and analysis on a high-quality RNA-Seq dataset of *Arabidopsis* under heat stress.

## 2. Results and Discussion

### 2.1. ASTool Can Accurately Identify AS Events

**Figure 1:**
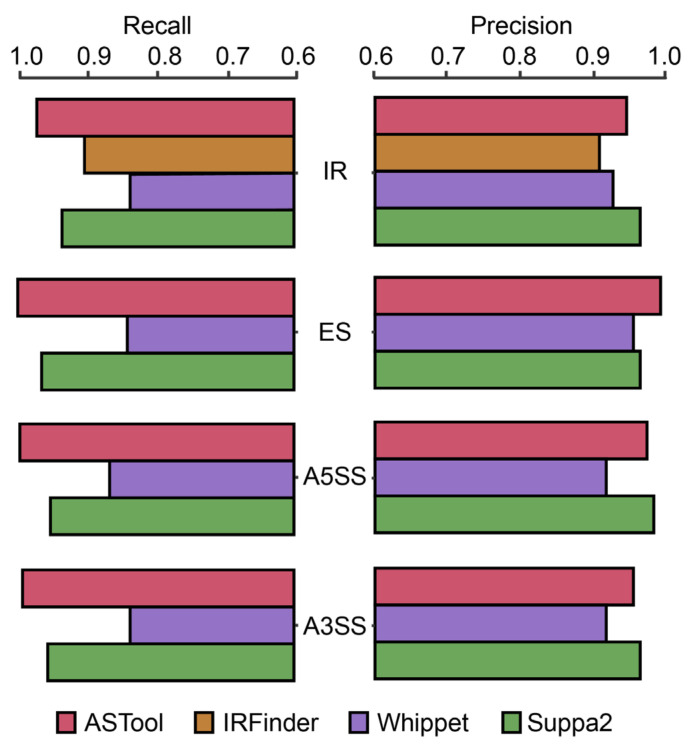
Performance comparison of ASTool with three existing tools. The performance comparison in detecting IR, ES, A5SS, and A3SS are based on the recall values (**left panel**) and the precision values (**right panel**) of different tools on the simulated RNA-Seq data.

We used the simulated RNA-Seq data to evaluate the performance of ASTool (see Section 3 for the methodology details, dataset preparation and performance measurements). In general, the proposed ASTool achieved an excellent performance in detecting AS events. The recall and precision values exceeded 95% in most cases. Specially, ASTool resulted in a recall value of 95.6%, 98.4%, 98.1%, and 97.7% in the identification of IR, ES, A5SS, and A3SS, respectively (Figure 1 and Table S1). In terms of precision, the corresponding values were 94.5%, 98.4%, 97.7%, and 96.0%, respectively (Figure 1 and Table S1). By jointly using the recall and precision metrics, we further compared the performance of ASTool against Whippet [24], IRFinder [23], and Suppa2 [27] on the same simulated data. In terms of recall and precision, ASTool showed a better or more competitive performance than the three existing tools (Figure 1). For the identification of IR events, ASTool showed considerably higher recall and precision values than Whippet and IRFinder, and it performed very similarly to Suppa2 regarding either the recall or precision. Regarding the ES event, ASTool showed a much better performance than Whippet, and reasonably increased performance compared to Suppa2. With respect to the identification of A5SS/A3SS events, ASTool showed a slightly better performance than Suppa2, and it still maintained higher recall and precision values than Whippet. We further tested ASTool on another simulated RNA-Seq dataset at a depth of around 30×, and the performance of ASTool only slightly decreased in comparison to the aforementioned results (Table S2). Therefore, the proposed ASTool should be applicable in the processing of RNA-Seq data with a relatively low sequencing depth.

In general, the more transcripts one gene contains, the more complicated the corresponding AS events are. Thus, we also compared different AS identification tools in processing genes with different numbers of transcripts. Comparatively, the different methods simultaneously revealed a better performance in processing genes with few transcripts (i.e., two transcripts) than those with multiple transcripts (i.e., more than two transcripts) (Figure S1). Interestingly, ASTool still achieved a robust and favorable performance in detecting AS events of genes containing multiple transcripts.

To evaluate the performance of ASTool more comprehensively, we also benchmarked the running time of different tools on the simulated RNA-Seq data (Table 1). Note that all methods were run on a DELL R930 computer server with 512 Gb of RAM and 10 Intel Xeon E5-4620 2.10 GHz CPU cores. When only a single thread was used, the computational speed of ASTool was comparable with IRFinder, but it was much lower than that of Suppa2 and Whippet (Table 1). The computational speed of ASTool was greatly accelerated when the number of running threads was appropriately increased. When 10 threads were allocated, the speed of ASTool improved nearly 7 times, which achieved a fully comparable level with the other tools. Considering that multiple threads are increasingly popular for current computer configurations, the computational speed of ASTool is generally rapid and competitive, which matched the need for large-scale RNA-Seq data analysis.

### 2.2. AS Events Detected by ASTool Are Consistent with Other Tools

**Figure 2:**
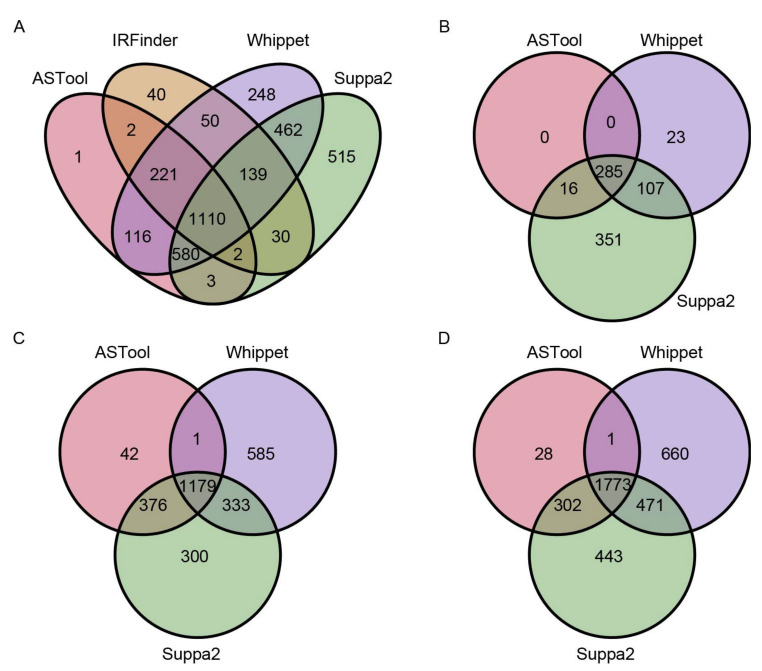
Venn diagrams of the four types of AS events detected by different tools. Overlapping relationships of the four types of AS events are displayed, including (**A**) IR events, (**B**) ES events, (**C**) A5SS events, and (**D**) A3SS events. The latter three types of AS events are undetectable in IRFinder.

To further compare the results of AS events, we compared the overlapping of AS events detected by different tools. We used IR and ES events labeled as “known” and “clean” by ASTool for the comparison with other tools (see Section 3 for the definitions of “known” and “clean”). Figure 2A shows the number of IR events detected by ASTool, IRFinder, Whippet, and Suppa2 on the simulated RNA-Seq data. Interestingly, only one IR event from ASTool could not be identified by the other tools. Likewise, ASTool also showed high consistency with the other tools in identifying ES/A5SS/A3SS events (Figure 2B–D). Moreover, we used real RNA-Seq data of *Arabidopsis* under heat stress (GSM2467113) to investigate the overlapping of these 4 tools, and the excellent consistency of ASTool with the other tools was observed again (Figure S2). It is worth noting that the IR event accounts for the largest proportion of the four AS events and the ES event accounts for the smallest proportion, regardless of whether the above simulated RNA-Seq data or the real RNA-Seq data were used. Therefore, ASTool can strictly detect AS events and provide reliable identification results.

### 2.3. ASTool Can Detect and Visualize Novel IR Events

**Figure 3:**
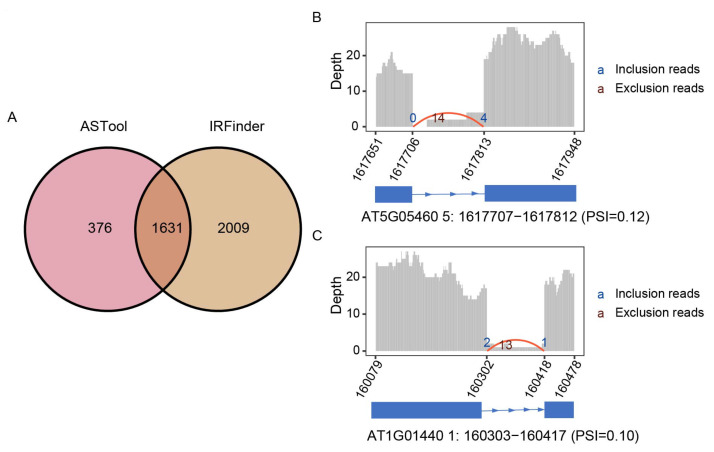
Novel IR event detection and visualization with ASTool. (**A**) Overlapping of novel IR events detected by ASTool and IRFinder. (**B**) Visualization of a potential IR event of gene AT5G05460 only identified by IRFinder. (**C**) Visualization of a potential IR of gene AT1G01440 only identified by ASTool. Genes were colored blue and plotted with rectangles representing exons and lines representing introns at the bottom. Bar chart represents the mapping depth (*y*-axis) of each locus, with the *x*-axis marked with positions of the start and end gene loci in the genome. Red and blue numbers indicate the number of inclusion and exclusion reads, respectively. Inclusion reads denote those reads mapped to the E1_I and I_E2 junctions while exclusion reads stand for those reads mapped to the E1_E2 junction.

We further used the real RNA-Seq data of *Arabidopsis* under heat stress (GSM2467113) to analyze and visualize novel IR events identified by ASTool. For comparison, novel IR events were also identified using IRFinder. Note that we selected introns labeled as “clean” by ASTool, and introns without warning for IRFinder. ASTool and IRFinder separately detected 2007 and 3640 IR events. Of them, a total of 1631 events overlapped (Figure 3A). Moreover, the visualization of the identified novel IR events was exemplified by two cases. In the first case, we focused on a potential IR event in AT5G05460, which ASTool failed to detect but was successfully identified by IRFinder. The AT5G05460 gene encodes *Arabidopsis* endo-β-n-acetylglucosaminidase (ENGase), with a full sequence length of 680 amino acids [28]. If the IR event occurs as detected by IRFinder, the gene will introduce a premature termination codon (PTC) at the 393th amino acid position, resulting in the loss of the following 287 amino acids. As shown in Figure 3B, the number of reads mapped to the junction connecting the left exon to the intron (i.e., E1_I junction) is zero, which is highly imbalanced with the junction connecting the intron to the right exon (i.e., I_E2 junction). Therefore, it is not assigned as a novel IR event in ASTool, although the calculated PSI value is 0.12. In the second case, we selected a potential retained intron in AT1G01440 that was successfully detected by ASTool but failed by IRFinder (Figure 3C). AT1G01440 encodes a GTP binding-related protein with a sequence length of 664 amino acids, playing an important role in stress resistance [29]. From the results of ASTool, although the number of reads mapping to the junction is relatively small, it can be assigned as an IR event since the inferred PSI value is equal to 0.10. Interestingly, the IR event will introduce PTC at the 572th position and result in 92 residues being missed at the C-terminal. In summary, ASTool can be used to detect and visualize novel retained introns intuitively, providing new hints to guide further experimental studies in deciphering gene functions.

### 2.4. Using ASTool to Analyze Arabidopsis AS Events in Response to Heat Stress

**Figure 4:**
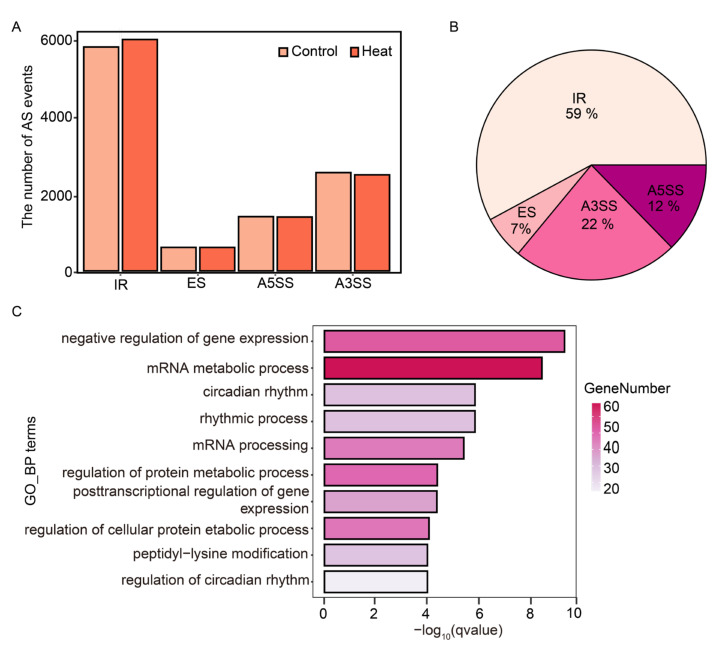
A case study of using ASTool in AS identification and analysis on real RNA-Seq data of *Arabidopsis* under heat stress. (**A**) The average number of the four identified AS types in the control and heat stress groups. (**B**) Distribution of the four differentially spliced AS events. (**C**) GO enrichment analysis of the identified (DSGs) on the biological process category.

To provide a case study revealing the functionality of ASTool, we selected three control samples (GSM2467110-GSM2467112) and three heat-stress-treated samples (GSM2467113-GSM2467115) from GSE94015 for analysis of the AS events of *Arabidopsis* in response to heat stress. First, we identified four major AS events in these samples under heat stress. Figure 4A shows the average numbers of these four major AS events in the control and heat stress groups. In general, the IR events are dominant in the heat stress group, which accounts for approximately 55% of the identified AS events, followed by A3SS, A5SS, and ES. Similarly, the numbers of the four AS events in the control group are close to those in the heat stress group.

We scrutinized differentially spliced events by comparing the PSI values of each AS event in the heat stress samples against the control samples. A differentially spliced event should meet the following two criteria: (1) |△PSI| ≥ 0.1; and (2) *p*-value based on Wilcoxon test ≤ 0.1. In all 4 AS types, a total of 2269 differentially spliced events were identified. Of them, differential IR events accounted for the highest proportion (59%), followed by A3SS (22%), A5SS (12%), and ES (7%) (Figure 4B). We referred to genes containing differentially spliced events as differential splicing genes (DSGs). In total, 1594 DSGs were identified in the selected RNA-Seq data. Moreover, we used the R package “clusterProfiler” [30] to perform gene ontology (GO) enrichment analysis of these DSGs under the category of biological process (BP). Note that the *Arabidopsis* GO terms in clusterProfiler were derived from Bioconductor AnnotationData Package (https://bioconductor.org/packages/release/BiocViews.html, org.At.tair.db (v3.14), accessed on 5 January 2022). Using the Benjamini–Hochberg method, the *p*-value was further modified to a q-value by controlling the false discovery rate (FDR). A total of 73 enriched GO terms were obtained (q-value < 0.05), and the top 10 GO terms are presented in Figure 4C. As expected, the results showed that DSGs were enriched in terms related to RNA splicing and post-transcriptional regulation of gene expression (Figure 4C). Interestingly, DSGs were also significantly enriched in items related to circadian rhythm regulation (Figure 4C). It has been reported that some molecular chaperones play critical regulatory roles in heat stress and circadian rhythm [31]. Thus, the enriched circadian rhythm-related functional terms may provide new hints to understand the functional roles of plant AS in dealing with heat stress. Collectively, the above application example indicates that ASTool can effectively and accurately identify differential splicing events, which can be used for large-scale plant RNA-Seq analysis.

## 3. Methods

### 3.1. Simulated RNA-Seq Data and Pre-Processing

**Figure 5:**
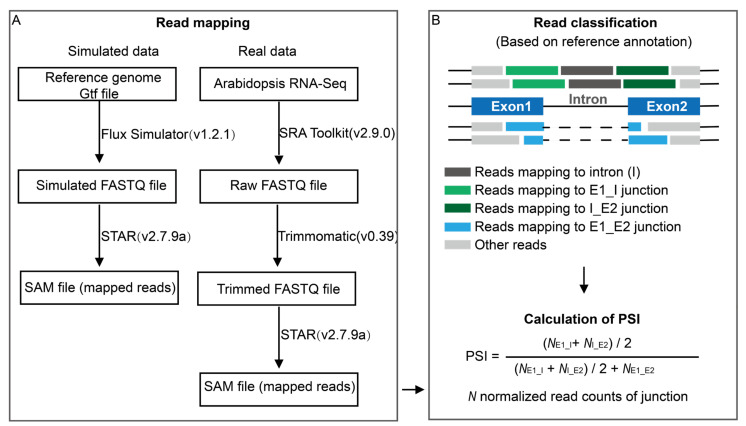
The preprocessing of RNA-Seq data and IR event identification with ASTool. (**A**) Data preparation. For simulated data, reads are simulated from *Arabidopsis* genome data. For real data, reads are directly from *Arabidopsis* RNA-Seq data downloaded from NCBI. As shown in panel A, the simulated/real data are then directly mapped to the reference genome with STAR. (**B**) IR detection and PSI calculation. SAM files are used to assign reads into 4 types of junctions (i.e., E1_I, I, I_E2, and E1_E2) (upper panel). Finally, the normalized read counts of 3 types of junctions (i.e., E1_I, I_E2, and E1_E2) are used to calculate PSI (lower panel).

To assess the performance of the developed ASTool, we used Flux Simulator (v1.2.1) [32] to simulate a set of 150 bp paired-end RNA-Seq data in FASTQ format. The reference genome and the corresponding annotation file of *Arabidopsis* were downloaded from EnsemblPlants (version: release 52) [33]. According to the genomic size, we simulated a total number of 60 million reads with a depth of about 70×. We used STAR (v2.7.9a) [34] to align reads to the reference genome with default parameters. The SAM file from STAR was converted to a BAM file using Samtools (v1.11) [35], and both were used for calculating PSI of AS events (Figure 5A).

### 3.2. Real RNA-Seq Data and Pre-Processing

To fully understand the functionality of ASTool, we selected 6 samples (GSM2467110–GSM2467115) from the stress-related 101 bp paired-end RNA-Seq data set GSE94015 [36] deposited in Gene Expression Omnibus (GEO) [37], and downloaded the corresponding raw data from NCBI Sequence Read Archive (SRA) [38], including flowering leaves of *Arabidopsis* after 3 h heat treatment and a control with 3 replicates in each condition. Note that the average sequencing depth of these samples was about 30×. The SRA files were converted to FASTQ files using fastq-dump from SRA Toolkit (https://github.com/ncbi/sratoolkit, accessed on 15 December 2021). Read trimming, read filtering, and adapter removal were conducted using Trimmomatic (v0.39) [39]. Then, trimmed reads were aligned to the reference genome using STAR with default parameters. The SAM files were sent to ASTool for further analysis (Figure 5A).

### 3.3. Estimation of PSI

We used Perl scripts to extract AS events from the reference gene annotation file and calculate their corresponding PSI values based on mapped reads from the SAM files. We used the IR event as an example to elaborate the calculation of PSI. Regarding the IR event, there are three junction types, including left exon-intron junctions (E1_I) and intron-right exon junctions (I_E2) and exon-exon junctions (E1_E2) (Figure 5B). A minimum overlap of m bp (8 bp is recommended) between aligned RNA-Seq reads and certain exons or introns should exist [40]. To meet this criterion, the exons or introns with lengths less than m bp are filtered out. For each junction, the aligned read counts should be normalized by the effective length (i.e., the number of unique mappable positions for RNA-Seq reads). The effective lengths of E1_I, I_E2, and E1_E2 junctions are associated with the length of RNA-Seq read (L) and m, which are L−2m + 1. The PSI value for each intron can be calculated through the following formula:

$$\mathrm{PSI}=\frac{0.5\;\times \;(N_{E1\_I}+N_{I\_E2})}{0.5\;\times \;(N_{E1\_I}+N_{I\_E2})+N_{E1\_E2}}$$

where *N*_E1_I_, *N*_I_, *N*_I_E2_, and *N*_E1_E2_ are the normalized read counts for junctions (Figure 5B). Similarly, the PSI values for the ES and A5SS/A3SS events can be calculated (Figure S3).

### 3.4. Comparison with Existing Tools

We compared ASTool with three existing tools (i.e., Whippet [24], IRFinder [23], and Suppa2 [27]) using simulated RNA-Seq data. In this work, only strictly defined AS events (see Figure S4 for the definitions of strict AS events) were used for method comparison. For each AS type, the strict AS events were generated from the most recent annotation files of *Arabidopsis*. The expression levels of isoforms generated from the Flux Simulator were used to calculate the true PSI of each AS event. For each AS event, we assumed that the expression level of intron-containing isoform is Ec and the expression level of intron-removed isoform is Er. Then, the true PSI = Ec/(Ec + Er). Regarding the IR/A5SS/A3SS events, a real PSI value ≥ 0.1 corresponded to a positive AS event while a real PSI value < 0.1 was regarded as a negative AS event. However, the true PSI value should be ≤ 0.9 (1−PSI ≥ 0.1) for the positive ES events, and the true PSI value should be > 0.9 (1−PSI < 0.1) for the negative ES events.

Regarding the AS identification, ASTool relied on the exon and intron information of different transcripts in the genome annotation file to assign “known” AS events. If an intron is a retained intron, the corresponding IR event is labeled as “known”. Similarly, if an exon is a skipped exon, the corresponding ES event is labeled as “known”. When a gene contains multiple transcripts, if an intron in one transcript does not partly overlap with the exons of other transcripts, it is assigned as a “clean intron”. The same principle is applied for the definition of “clean exon”. When conducting performance comparison, we only take the AS events marked by ASTool as “known” and containing “clean intron/exon” into account (Figure S5). ASTool yields a PSI value for each AS event. Likewise, we can assign the AS event as a predicted positive or negative AS event. Specially, a PSI value ≥ 0.1 corresponded to a predicted positive AS event while a PSI value < 0.1 was regarded as a predicted negative AS event in processing the IR/A5SS/A3SS events. Conversely, the PSI value should be ≤ 0.9 (1−PSI ≥ 0.1) for the predicted positive ES events, and the true PSI value should be > 0.9 (1−PSI < 0.1) for the predicted negative ES events.

The calculation of PSI using the three existing methods was implemented by downloading the corresponding source codes and running them in our local machine. Whippet (v0.11.1) (https://github.com/timbitz/Whippet.jl, accessed on 5 December 2021) was applied to calculate PSI values based on sorted BAM files. IRFinder (IRFinder-1.2.5) was downloaded from Github (https://github.com/williamritchie/IRFinder, accessed on 10 December 2021), and the default parameters were applied to calculate PSI values from FASTQ files. To ensure enough sequencing depth for AS events, we filtered the introns annotated as “LowCover”. Suppa2 (v2.3) used Python scripts to calculate PSI values according to the expression of transcripts, which was downloaded from https://github.com/comprna/SUPPA, accessed on 13 December 2021.

We used two measurements (i.e., recall and precision) to evaluate the performance of different AS identification tools, which are defined as:

$$\mathrm{Recall}=\frac{TP}{TP+FN}$$

$$\mathrm{Precision}=\frac{TP}{TP+FP}$$

where *TP*, *FP* and *FN* are true positives (i.e., the number of correctly identified positive AS events), false positives (i.e., the number of negative AS events identified as positive AS events), and false negatives (i.e., the number of positive AS events identified as negative AS events), respectively. It should be emphasized that the benchmark dataset (i.e., the positive and negative AS events) was obtained from the true PSI values inferred from the simulated RNA-Seq data.

## 4. Conclusions

In this work, we developed an AS identification tool called ASTool to detect and analyze the AS events from plant RNA-Seq data. Compared with several popular existing tools, ASTool showed a competitive performance on simulated RNA-Seq data. To fully present the advantage of ASTool, the functionality of different tools is also summarized in Table 2. We have made the Perl scripts and user manual of ASTool freely available at http://zzdlab.com/ASTool/index.php (accessed on 10 December 2021) or https://github.com/zzd-lab/ASTool (accessed on 10 December 2021). We anticipate that the proposed ASTool can become a useful and competitive tool for the analysis of AS events from large-scale plant RNA-Seq data, and thus accelerate the deciphering of the functional role of AS in the plant co-transcriptional regulatory mechanism.

## Figures and Tables

**Table 1:** Computational time required by different tools in the detection of AS events in the simulated data.

Tools	Single Thread (min)	Ten Threads (min)
ASTool	160.4	23.5
Whippet	32.0	NA ^a^
IRFinder	154.4	31.3
Suppa2	16.1	15.4

^a^ NA means the option of multiple threads is not available.

**Table 2:** Functional options of ASTool and three existing tools in the detection of different AS events.

Classification	Tools	Detection of Four Main AS Types	Detection of Non-Strict AS Events	Detection of Novel IR Events
Exon-based	ASTool	√	√	√
	Whippet	√	√	×
	IRFinder	×	√	√
Transcript-based	Suppa2	√	×	×

## Supplementary Materials

The following supporting information can be downloaded at: https://www.mdpi.com/article/10.3390/ijms23084079/s1.
