# Supplementary material for: ASTool: An Easy-to-Use Tool to Accurately Identify Alternative Splicing Events from Plant RNA-Seq Data
Source: Int J Mol Sci. 2022 Apr 7;23(8):4079. doi: 10.3390/ijms23084079 (PMC9031537; doi:10.3390/ijms23084079)
Supplement: Supplementary file 1 [file ijms-23-04079-s001.zip › ijms-1617194-Supplementary Materials.pdf]

Table S1. The Recall and Precision values of ASTool in comparison with the other three tools.

AS Type	Tools	Recall	Precision
IR	ASTool	**95.6%**	**94.5%**
	IRFinder	88.6%	91.4%
	Whippet	82.2%	93.0%
	Suppa2	91.9%	96.9%
ES	ASTool	**98.4%**	**100.0%**
	Whippet	82.3%	95.5%
	Suppa2	94.9%	97.0%
A5SS	ASTool	**98.1%**	**97.7%**
	Whippet	84.9%	92.1%
	Suppa2	93.6%	98.8%
A3SS	ASTool	**97.7%**	**96.0%**
	Whippet	81.9%	92.2%
	Suppa2	94.0%	96.8%

Table S2. The Recall and Precision values of ASTool at two different sequencing depths.

AS Type	Depth	Recall	Precision
IR	70x	95.6%	94.5%
	30x	94.3%	94.1%
ES	70x	98.4%	100.0%
	30x	96.8%	96.8%
A5SS	70x	98.1%	97.7%
	30x	97.5%	97.9%
A3SS	70x	97.7%	96.0%
	30x	95.1%	95.5%

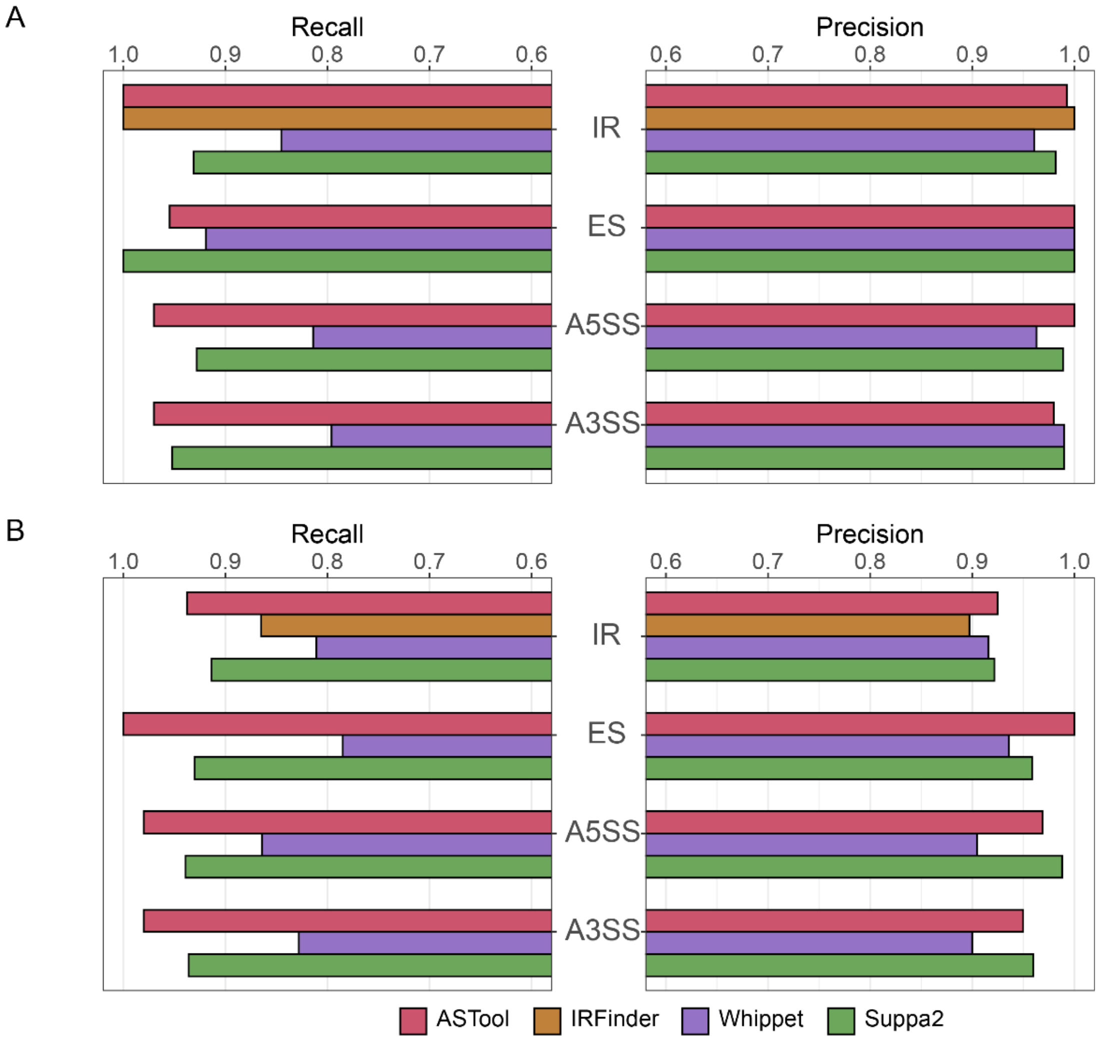

Figure S1. The performance comparison of the four tools in terms of the number of transcripts contained in the genes. (A) The performance of the four tools when the gene contains two transcripts. (B) The performance of the four tools when the gene contains more than two transcripts.

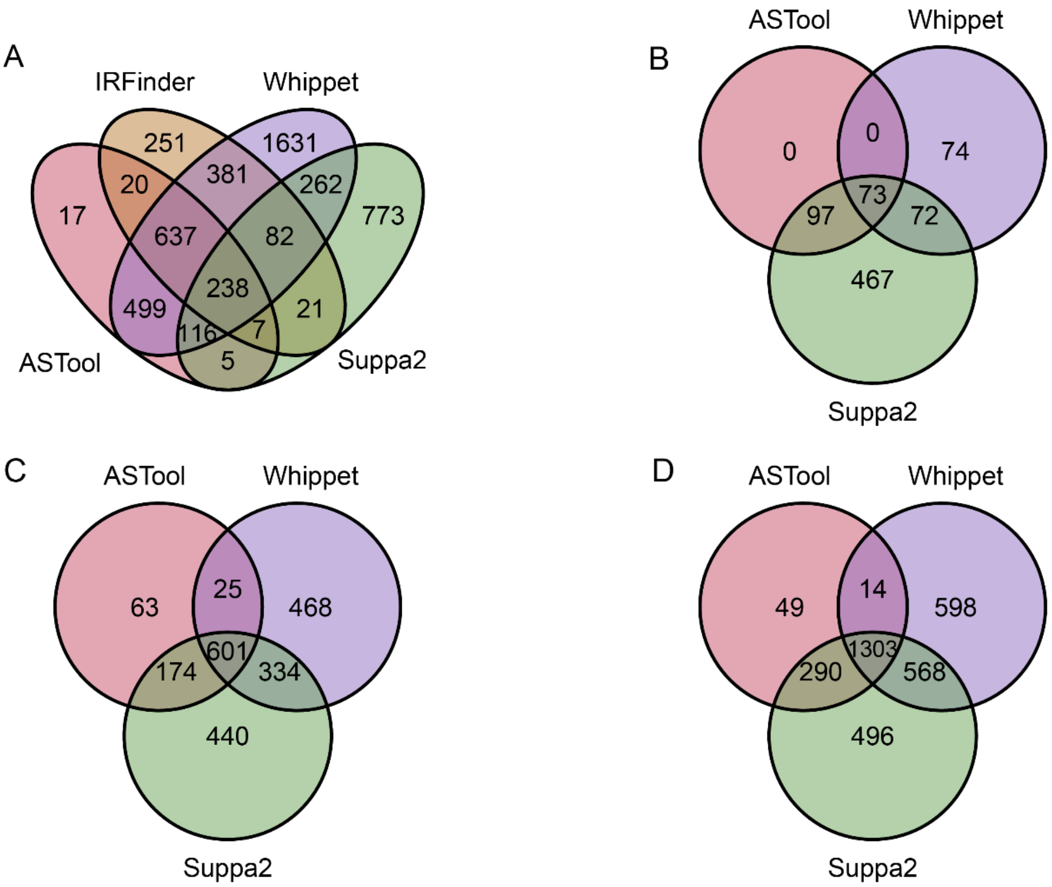

Figure S2. Overlapping of AS events detected by different tools in real RNA-Seq data. (A)The number of IR events detected by ASTool, IRFinder,Whippet, and Suppa2. (B) The number of ES events detected by ASTool, Suppa2 and Whippet. (C) The number of A5SS event detected by ASTool, Suppa2, and Whippet. (D) The number of A3SS events detected by ASTool, Suppa2, and Whippet.

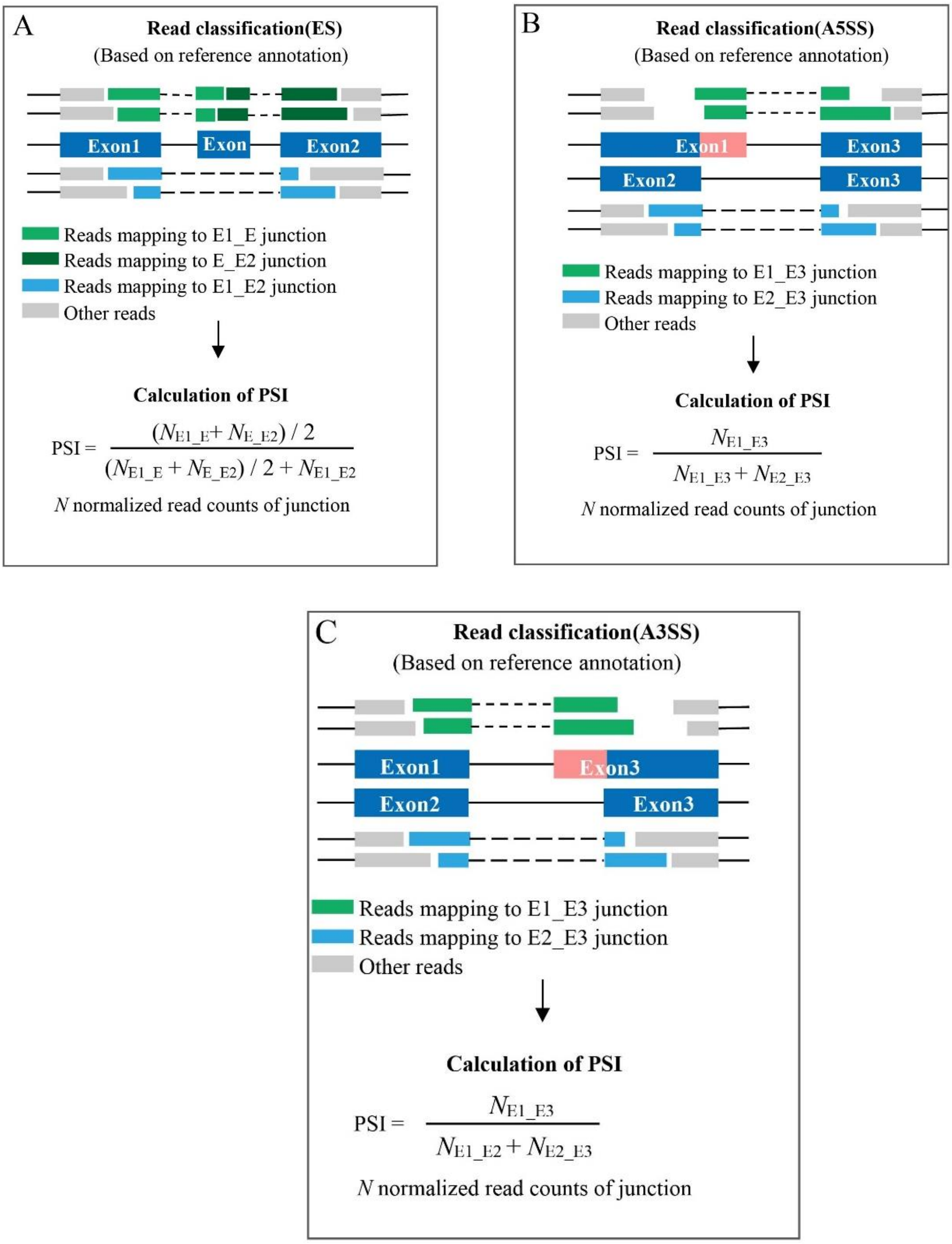

Figure S3. Schematic diagram showing the calculation of PSI of the ES (A), A5SS (B) and, A3SS (C) events.

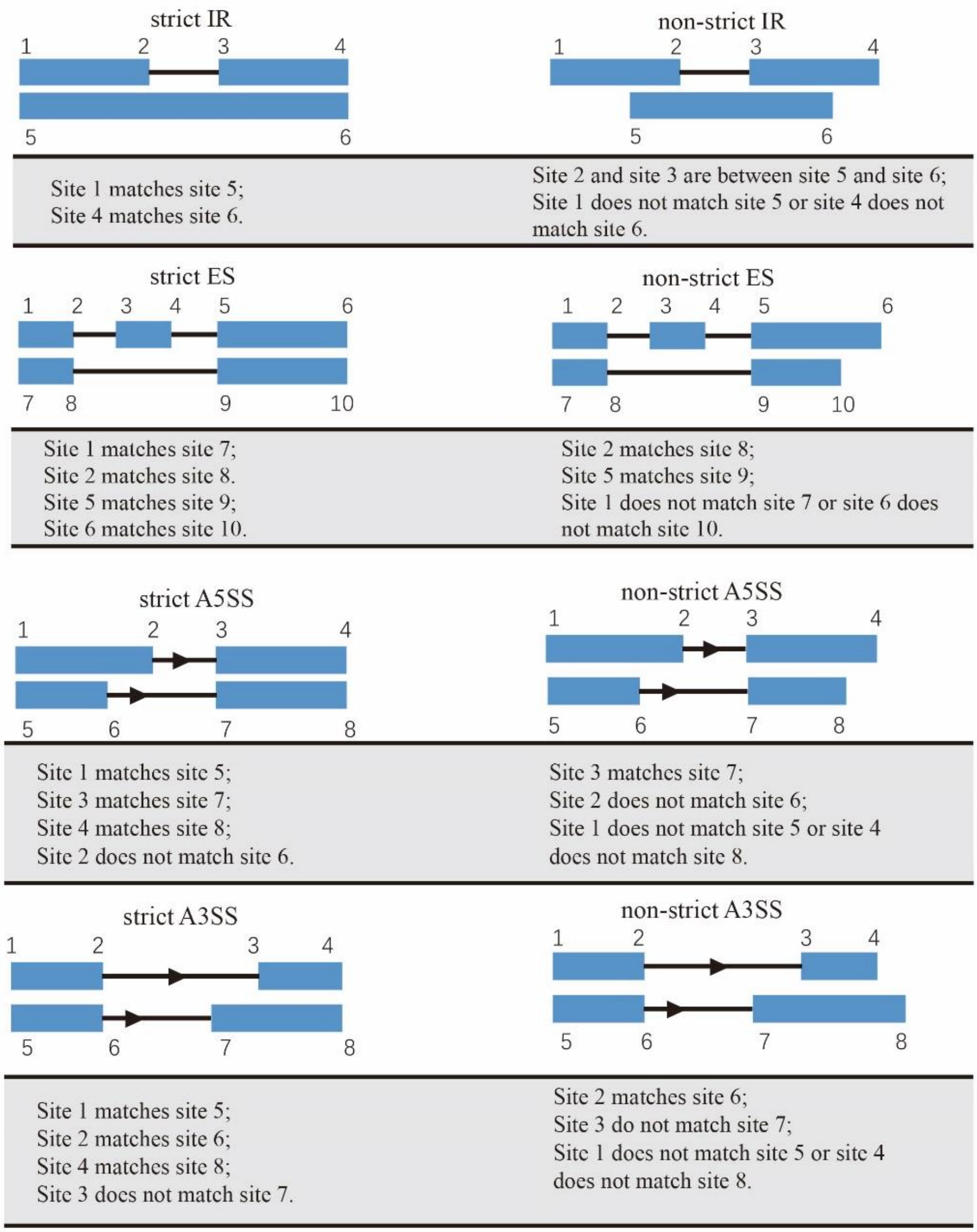

Figure S4. Schematic diagram of strict (left) and non-strict (right) AS events. The four types of AS events from top to bottom are IR, ES, A5SS, and A3SS.

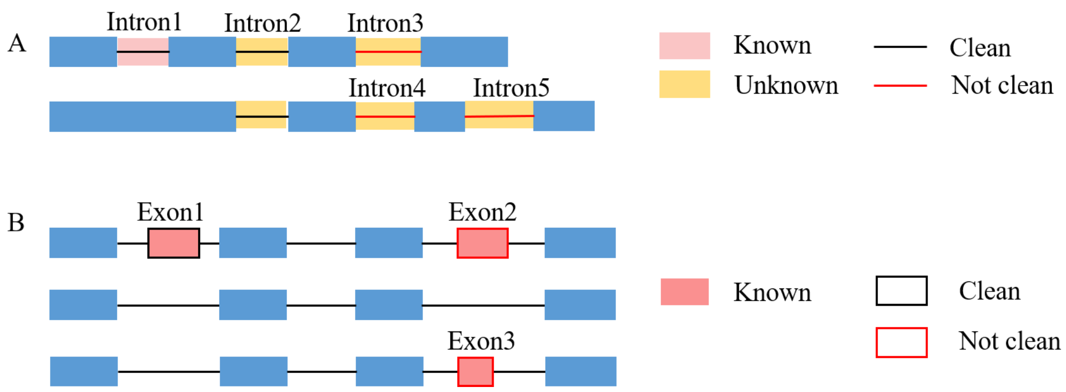

Figure S5. Classification of introns and exons. (A) Classification of different types of introns. Intron1 is a retained intron without overlapping with other exons or introns, so it is labeled as "Known" and "Clean". Intron2 is not annotated as an IR event and has no overlap with other exons/introns, so it is annotated as "Unknown" and "Clean". Intron3, Intron4, and intron5 have overlap with other introns/exons, these are annotated as “Not clean” introns. (B) Classification of different types of exons. Exon1 is a skipped exon labeled as "Known" and "Clean". The other two “Known” Exon2 and Exon3 are labeled "Not Clean" because the two exons partially overlap and cannot accurately calculate the PSI values of the two ES events.
